# How Fitness Reduced, Antimicrobial Resistant Bacteria Survive and Spread: A Multiple Pig - Multiple Bacterial Strain Model

**DOI:** 10.1371/journal.pone.0100458

**Published:** 2014-07-09

**Authors:** Kaare Græsbøll, Søren Saxmose Nielsen, Nils Toft, Lasse Engbo Christiansen

**Affiliations:** 1 Department of Applied Mathematics and Computer Science, Technical University of Denmark, Lyngby, Denmark; 2 Department of Large Animal Sciences, University of Copenhagen, Frederiksberg, Denmark; 3 National Veterinary Institute, Technical University of Denmark, Frederiksberg, Denmark; Charité, Campus Benjamin Franklin, Germany

## Abstract

More than 30% of *E. coli* strains sampled from pig farms in Denmark over the last five years were resistant to the commonly used antimicrobial tetracycline. This raises a number of questions: How is this high level sustained if resistant bacteria have reduced growth rates? Given that there are multiple susceptible and resistant bacterial strains in the pig intestines, how can we describe their coexistence? To what extent does the composition of these multiple strains in individual pigs influence the total bacterial population of the pig pen? What happens to a complex population when antimicrobials are used? To investigate these questions, we created a model where multiple strains of bacteria coexist in the intestines of pigs sharing a pen, and explored the parameter limits of a stable system; both with and without an antimicrobial treatment. The approach taken is a deterministic bacterial population model with stochastic elements of bacterial distributions and transmission. The rates that govern the model are process-oriented to represent growth, excretion, and uptake from environment, independent of herd and meta-population structures. Furthermore, an entry barrier and elimination process for the individual strains in each pig were implemented. We demonstrate how competitive growth between multiple bacterial strains in individual pigs, and the transmission between pigs in a pen allow for strains of antimicrobial resistant bacteria to persist in a pig population to different extents, and how quickly they can become dominant if antimicrobial treatment is initiated. The level of spread depends in a non-linear way of the parameters that govern excretion and uptake. Furthermore, the sampling of initial distributions of strains and stochastic transmission events give rise to large variation in how homogenous and how resistant the bacterial population becomes. Most important: resistant bacteria are demonstrated to survive with a disadvantage in growth rate of well over 10%.

## Introduction

Reducing the emergence and spread of antimicrobial resistance is a major challenge, and the use of antimicrobials in production animals is considered to be an important contributor to resistance development [1], [2]. Still, the use of antimicrobials is necessary in livestock production to avoid compromising animal health and welfare.

Antimicrobial resistance in Denmark is monitored through the Danish Integrated Antimicrobial Resistance Monitoring and Research Programme (DANMAP), which has shown consistently high levels (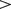
30% of the bacterial population in the past five years) of tetracycline resistant *Escherichia coli* in pigs and pork [3]. This indicates that tetracycline resistant bacterial strains are endemic in the pig population. Pig production also accounts for approximately 80% of the veterinary use of antimicrobials in Denmark, with tetracycline being the drug most frequently used [3]. Tetracycline is also frequently used in humans [3], and therefore is an antimicrobial that is of interest in both the human and veterinary sectors. Antimicrobials used in Danish pig production are often distributed as therapeutic flock treatment, and this is legal in the weaner facility when minimum of 25% of pigs in a section experience clinical diarrhea. This form of treatment results in most Danish pigs receiving multiple treatments of antimicrobials during their lifetime. Furthermore, flock treatments are given through feed or water, which may lead to high concentrations of antimicrobials being present in the intestinal system of pigs.

The World Organisation for Animal Health (OIE) promotes prudent use of antimicrobials through a set of reviewed guidelines [4]. Such guidelines continuously require updates, and mathematical models are considered to be a valuable tool in the battle against antimicrobial resistance [5], [6]; e.g. to determine optimal dosing strategies [7]. The high and persistent levels of some types of resistant strains such as tetracycline resistant *E. coli* need to be reflected in the modeling of these.

The fitness cost of antimicrobial resistance has been investigated in a number of studies, and while it is clear that achieving resistance can have a fitness cost in terms of e.g. bacterial growth rate, it has also been shown that this fitness cost is gradually reduced with time [8]–[10]. This adaptation towards similar growth rates allows resistant strains to survive for long periods, even without the selective pressure provided by an antimicrobial treatment.

Furthermore, it has been demonstrated that the total composition of strains in the gut may influence the ability of resistant strains to grow, and that treatment with antimicrobials affects the balance of the bacteria [11], [12].

Modeling of bacterial growth in response to antimicrobials has been often been focused on single bacteria strains with large emphasis on the response to antimicrobials [13], [14] and not on the ability of the system to have multiple coexisting strains. Furthermore, the contribution of fitness costs, transmission, and excretion in an environment with multiple competing bacterial strains has not previously been assessed in a pig production unit [15].

Thus, the objective of this study was to create a model with the coexistence of multiple strains with different responses to antimicrobials within each pig, in order to assess how pigs' excretion and within-herd spread of multiple bacterial strains affect the level of antimicrobial resistance following antimicrobial treatment. The model is generic so different bacteriostatic drugs can easily be implemented; in this paper we use tetracycline as a model drug.

## Methods

The developed model includes the growth of multiple bacterial strains in multiple pigs, along with the modeling of the transmission of strains between pigs sharing pens.

The growth of strains in the model is affected by antimicrobial concentration, and the total bacterial count. Transmission of strains is described by excretion of bacterial content from the individual pigs to the environment, and then uptake of a fraction of the total excreted bacterial material. When a new strain enters a pig in low bacterial numbers, or when an existing strain reduced to a low number by competition, there is imposed a risk of being removed from the individual pig.

The growth, excretion, and uptake of strains were modeled deterministically, due to the very large bacteria count. This was the most feasible with respect to computing time and the loss of variance from this approach is not large compared to the variance introduced from having unique distributions of strains in individual pigs. The initial distribution of strains in the individual pigs and the growth parameters of strains were drawn from distributions that best describe the parameters. The removal of strains from the pigs was modeled probalistic, so that both the transmission of a new strain to a pig, and the removal of slow growing strains are stochastic events.

### Assumptions

The model rests on the following assumptions, the influence of which is discussed more thoroughly in the discussion section.

- Strains are fully identified and described by their growth rate and how this depends on the concentration of antimicrobials.
- Strains are considered to be unique and independent of each other. E.g. there is not a resistant and susceptible version of the same strain *per se*.
- The emergence of resistance to tetracycline is negligible compared to the growth of existing resistant strains.
- Pigs are assigned to a pen and no movement of pigs between pens is considered during the model period.
- Within the pen, fecal matter is randomly mixed so that the pigs that share the pen experience a similar uptake/transmission of bacterial matter.
- Disease is not a special condition in the model, e.g. with respect to behavior of microbial intestinal flora and antimicrobial uptake and effect, and therefore, all pigs behave similarly.
- No pathogenic strains are identified and no immune response is modeled.
- The treatment of the animals is through a five-day treatment of the flock treatment the pigs are in, and all of the animals received the same dose. The resulting concentration in the intestines was set to 40 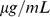 constantly during the treatment period.
- The transmission between pens is much smaller than within pen, and is therefore neglected.
- The growth parameters of the bacterial strains do not change, specifically the response to antibiotics are constant, i.e. spontaneous loss of resistance does not occur.
- The system is considered closed for the duration of simulation so new strains are not introduced during the period of interest.
- Antimicrobial treatment is the only intervention during the period of interest.

### Model formulation

**Figure 1:**
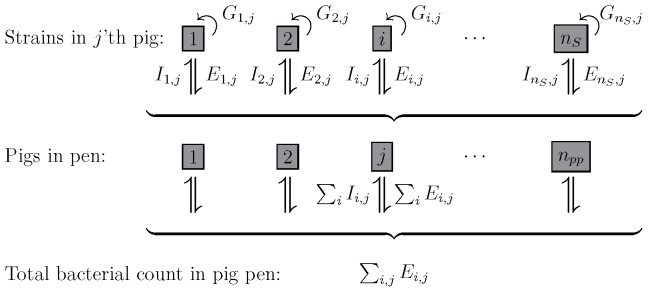
The model structure. For each pig the growth, 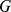, excretion, 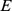, and intake, 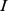, of 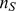 unique bacterial strains are modeled. Excreted bacterial material is summed up pen-wise, and a fraction will be taken back in by the 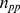 pigs sharing the pen. Excretion is driven by the excretion rate, 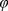; while transmission is described by the uptake fraction from the environment, 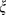; furthermore, bacterial strains in amounts below a cutoff value, 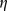, in each individual pig have a probability of being eliminated.

The model for multiple strains in multiple pigs was described by the set of differential equations given by:
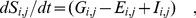
(1)where 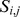 denotes the bacterial count of the 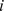
'th strain of bacteria in the 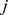
'th pig's intestines; 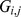 is the growth of bacteria; 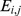 describes the excretion of bacteria to the pen environment; 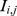 describes the intake of strains from the faeces excreted by all pigs; and 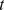 is time. Furthermore, the risk of removal for a strain is given by the term 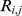 which is not expressed in continuous time. The model is presented in figure 1; please note that 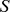 represents any strain of bacteria not only susceptible strains.

The model of growth in individual pigs, 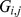 comprises multiple parts. First, the growth rate of bacteria, 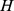, is modeled using a Hill-type equation to describe the influence of antimicrobials [16]–[18]: 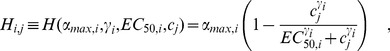
(2)where 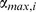 is the growth rate of the 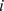
'th strain when no antimicrobial is present; 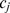 is the antimicrobial concentration in the 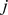
'th pig; 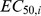 is the antimicrobial concentration at which the bacteria grow at half the rate as 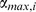; and 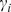 is the ‘Hill-coefficient’, which determines the steepness of the curve around 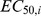.

The growth rate equation (2) is then used in differential equations that describe the competitive growth of strains in one pig: 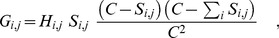
(3)where 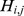 is short notation for 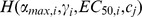, the growth rate for the 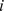
'th strain as described in equation (2) with the antimicrobial concentration present in the 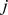
'th pig; 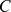 is the bacterial carrying capacity for each pig's intestines; and 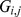 expresses the total growth term per strain per pig. Differential equations with a first order term that includes the carrying capacity, 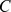, have been used extensively throughout bacterial population modeling [6], [15]. Using a second order term including carrying capacity is necessary to ensure restricted growth, which leads to co-existence of multiple strains as opposed to one strain outcompeting the rest.

The excretion of strains from the pigs' intestines is described by: 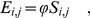
(4)where 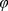 is the rate at which bacteria is excreted from the intestines.

The intake of strains from other pigs in the pen is defined as: 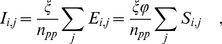
(5)where 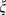 is the fraction of bacteria that comes back in from the environment. The environment is defined by the combined excretion from the pigs that share a pen. The equation is then normalized by the number of pigs per pen, 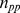, so that the intake of feces does not increase with an increased pen size. Examples of the full equations are included in File S1.

Removal, 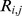, of a bacterial strain, 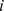, from the 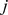
'th pig is an event described by the probability: 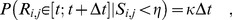
(6)so that there is a probability 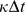 that the count of strain 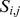 becomes zero within a given time interval, 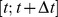, given that the bacterial count, 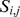, is below 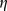. This term can be thought of as the probability of surviving in the gut when entering from the external environment, or losing the competition to strains with higher growth rates.

The transmission of bacteria as described by equations (5) and (6) can best be categorised as direct transmission with complete random mixing, because all pigs receive the same amount of bacteria from the environment, and the environment is not modeled explicitly over time. Even though pigs receive the same amount of bacteria; establishing a new strain in a pig is a stochastic process that depends on the strain surviving the repeated risk of removal (equation 6).

It was assumed that conjugation and other means of transmission of resistance between strains have little contribution to the overall level of resistance compared to the growth. Therefore the strains in the model do not alter their resistance levels (e.g. growth parameters describing 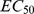).

The parameters used to describe this model were selected to be as close as possible to describing *in vivo* events. However, many of these parameters are given with large uncertainties and therefore we explore the outcomes for range of parameter values, as described in the following two sections.

### Selection of strains

The fraction of antimicrobial resistant strains in the population was set to 40% in this study, approximately corresponding to the observed values for tetracycline resistant *E. coli* in DANMAP (2012) [3].

Growth parameters were sampled from distributions: 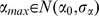, where 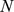 is the normal distribution, 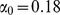, and 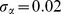. The value of 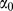 was taken from the literature [19], the default 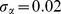 was set so that most strains survive the excretion/removal process; with the rationale that the pigs simulated have already lived for some period of time, which would have removed the strains of very low growth rates.

Strains susceptible to antimicrobials had 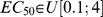 and 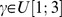, for antimicrobial resistant strains 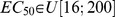 and 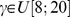, with 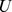 being the uniform distribution. The cut-off in sampling of 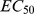 to define resistant and susceptible strains is in line with international standards of MIC values [20].

All growth parameters' distributions were unchanged in the model runs, except 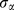, which was varied in some runs to test the maximum loss of fitness that a strain can endure before being in danger of extinction. When varying 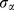, growth rates were not sampled, but chosen as the 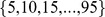 percentile of the normal distribution with the selected 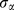.

### Model runs

The model was initiated by selecting the maximum number of strains in each pig, 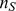, and assigning growth parameters to these strains as described in the previous section. For each pig initial presence of a strain was determined by a Bernoulli trial with probability 0.5 per strain per pig; on average any pig would therefore start with 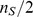 unique strains. Furthermore, the number of pigs per pen, 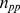, the number of pens, 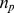, the length of the simulation in days, and the time and dose of antimicrobial treatment, if any, were declared.

To test the influence of excretion rate, 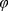, uptake fraction from the environment, 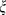, and the number of strains, 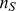, on the time to reach a stable level of the gut floras of pigs in the pen, a combination of three values of each of these parameters were tested.

In runs where parameters were not varied, the values were set to the same as were found in the literature: The default number of strains 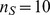 [21], and 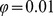 [19]. The default values of 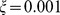 and 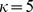 (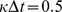) were not readily available from the literature and were therefore guesstimated. For the antimicrobial treatment, the dose present in the intestinal gut was assumed to be 40 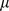 g/mL, and all of the treatments lasted for five days (in case of repeated treatments two times five days). The carrying capacity was set to 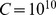.

The number of pens was typically 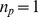, which did not affect the results as pens were treated independently in the model. The number of pigs per pen was set to 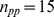 for all shown data. Some graphs were produced as the mean for a number of runs, this is indicated by the number of repeats 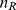.

Table 1 displays which parameter values were used to generate which figures.

**Table 1:** Parameter values and the figures they are used in.

Symbol		Unit	Value	Figures	Values	Figures	Description
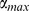		1/hour	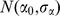	-	-	-	Maximum growth rate
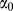		1/hour	0.18	2–8	-	-	Mean of maximum growth rate
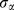		1/hour^2^	0.02	2–7	0.01  {1,2, 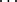 ,10}	8	Variance of maximum growth rate
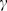	(susc)	-	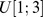	2–8	-	-	Hill-coefficient, susceptible strains
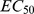	(susc)	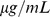	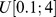	2–8	-	-	Half effect concentration, susceptible strains
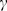	(res)	-	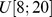	2–8	-	-	Hill-coefficient, resistant strains
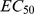	(res)	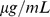	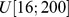	2–8	-	-	Half effect concentration, resistant strains
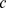		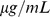	40	3,6–7	-	-	antimicrobial concentration
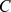		-	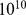	2–8	-	-	Carrying capacity
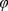		-	*	2–3,5,7	10^−3,−2,−1^	4,6,8	Excretion rate
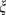		-	*	2–3,5,7	10^−4,−3,−2^	4,6,8	Uptake fraction
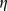		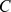	10 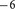	2–7	10^−6,−4.3,−3^	8	Removal cut-off
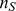		-	10	1,6–8	5,10,20	4–5	Max number of strains per pig
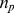		-	1	2–8	-	-	Number of pens
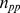		-	15	2–8	-	-	Number of pigs per pen
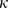		1/hour	5	2–8	-	-	Average number of removal events
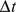		hour	0.1	2–8	-	-	Timestep in the model

*Various values: See figure legends for values.

To ensure that runs were comparable when parameters were changed, the sampling of the growth rates was given a seed so that the strains were the same for the different parameter sets tested; and the same composition of strains were used when comparing runs with and without antimicrobials.

The model was written in R version 2.15 ("Roasted Marshmallows") [22], all data was also analysed and plotted using R.

## Results

**Figure 2:**
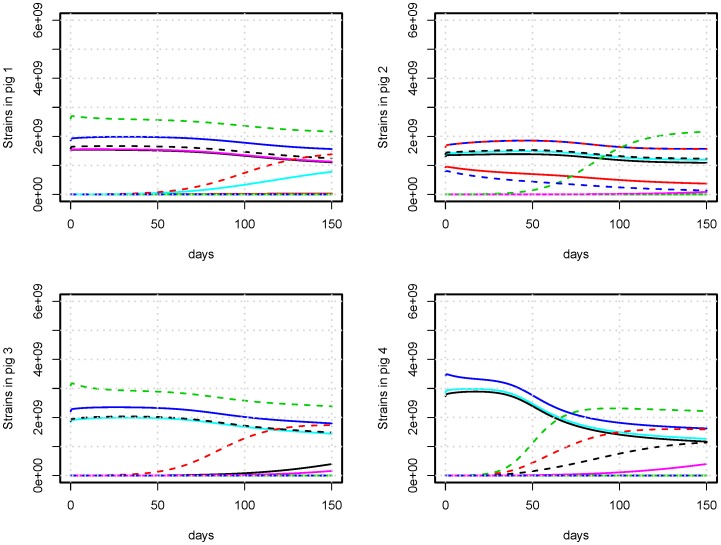
Bacterial composition of the gut of untreated pigs. An example of the bacterial count of strains in four pigs with no AMT. Unique strains are identified by different color. Resistant strains are identified with dashed lines. The bacterial populations are stable on the time-scale of days, and the transmission of strains from other pigs happen over a time-scale of months.

**Figure 3:**
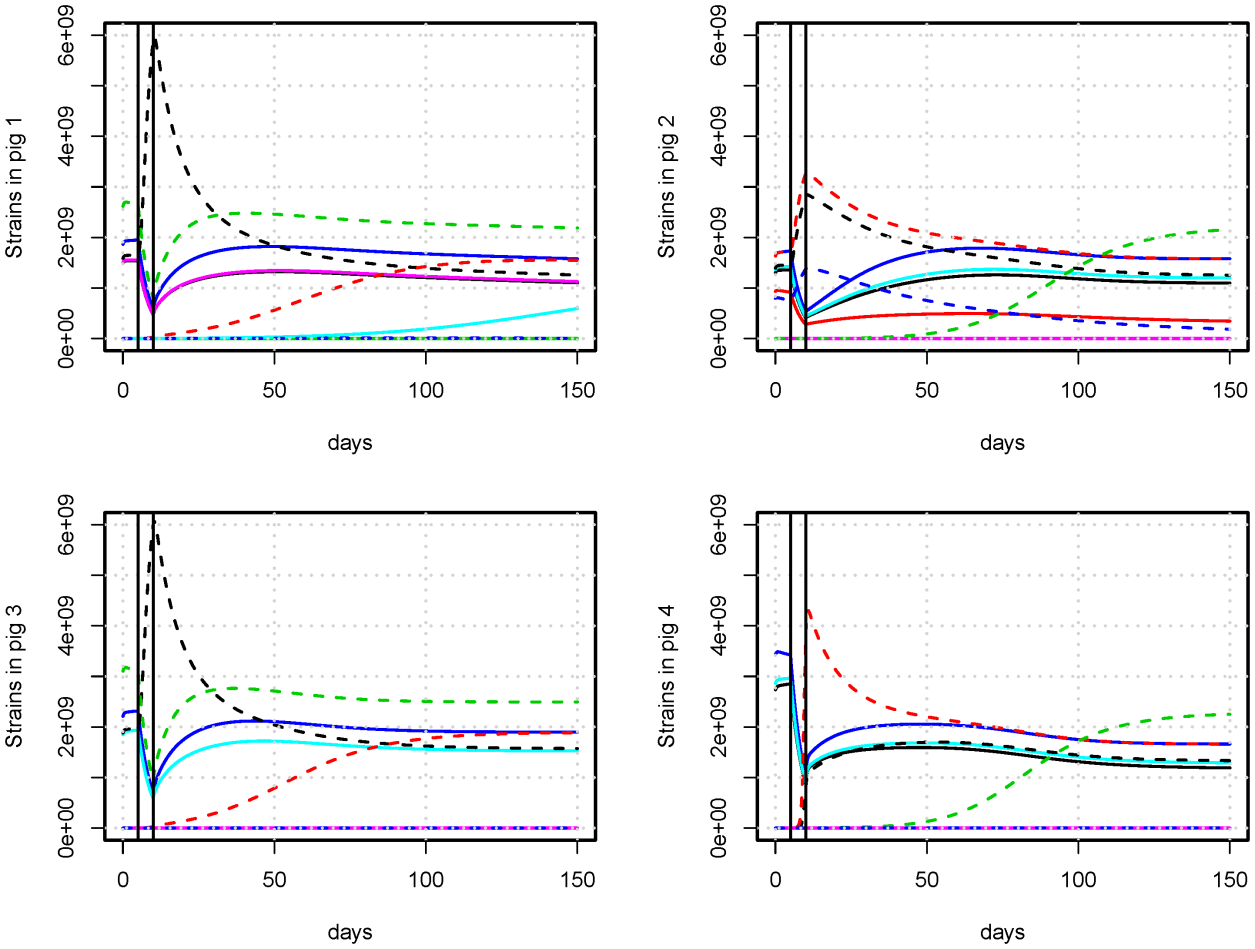
Bacterial composition of the gut of pigs treated with antimicrobials. An example of the count of strains in four pigs with AMT between day 5 and 10. Unique strains are identified by different colors. Resistant strains are identified with dashed lines. Antimicrobials are administered in the time between vertical black lines given as an effective concentration of 40 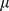
g/mL in the intestinal tract, which is so high that some strains labeled resistant (i.e. the green) also experience a decline. Compared to figure 2 with no antimicrobial treatment, resistant strains spread throughout the population on a time-scale of days, and an increase in resistant bacteria for months following treatment (see also figure 6) can be observed. The strains used in figure 2 and 3 are identical, only the use of antimicrobials is different.

**Figure 4:**
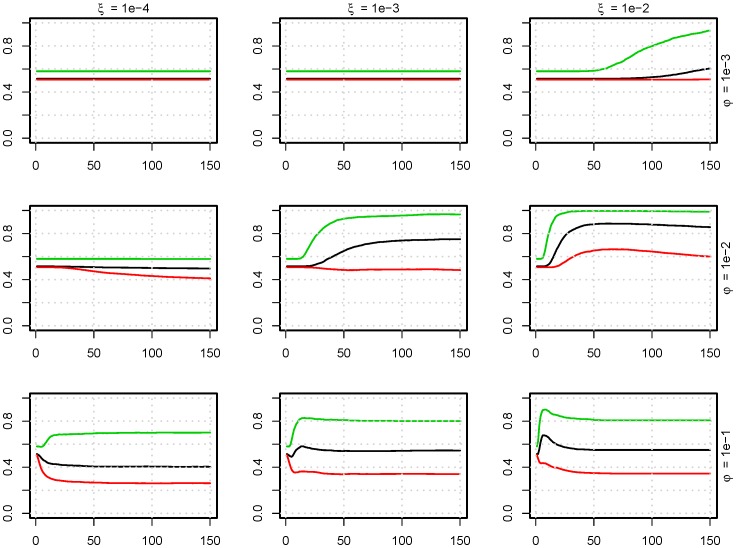
Level of homogenization of bacterial strains in a pig pen. The mean fraction of strains (y-axes) that is present in a pig (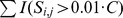 / 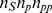) as a function of time [days] (x-axes), with no antimicrobial treatment. The colors indicate the number of strains in the model: green  = 5 strains, black  = 10 strains, and red  = 20 strains. This is modelled for nine parameter sets of 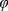 (excretion rate) and 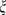 (uptake fraction from the environment). (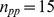, 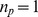, 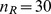).

**Figure 5:**
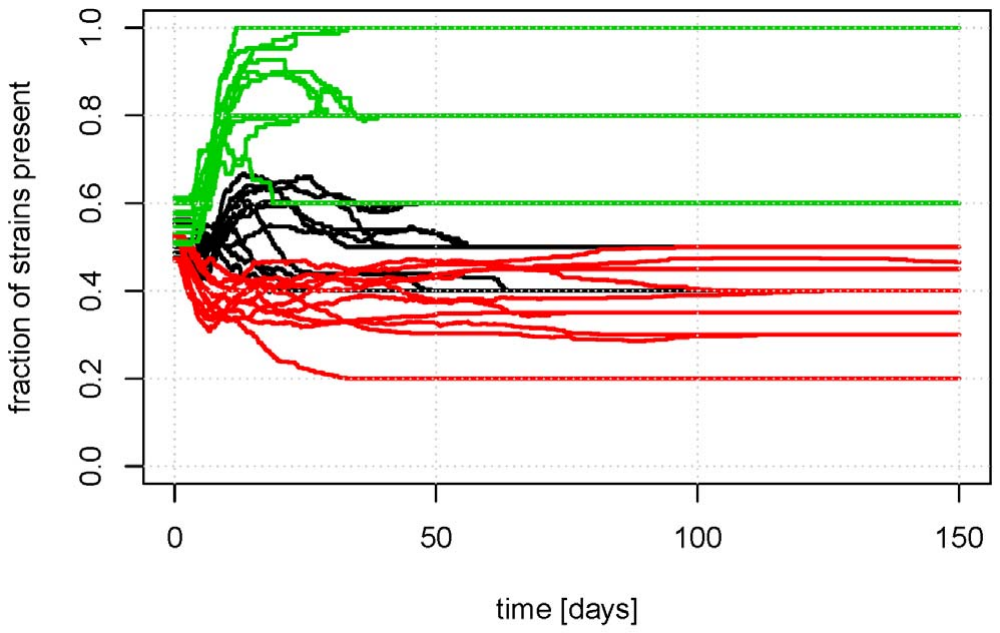
The variation of the repeats from figure 4. The mean fraction of strains that is present in a pig (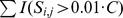 / 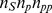) as a function of time with no antimicrobial treatment is shown for ten repetitions. The colors indicate the number of strains in the model: green  = 5 strains, black  = 10 strains, and red  = 20 strains. The leveling out after some time indicates that some strains have been completely eradicated from the pen population. (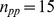, 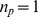, 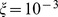, 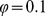).

**Figure 6:**
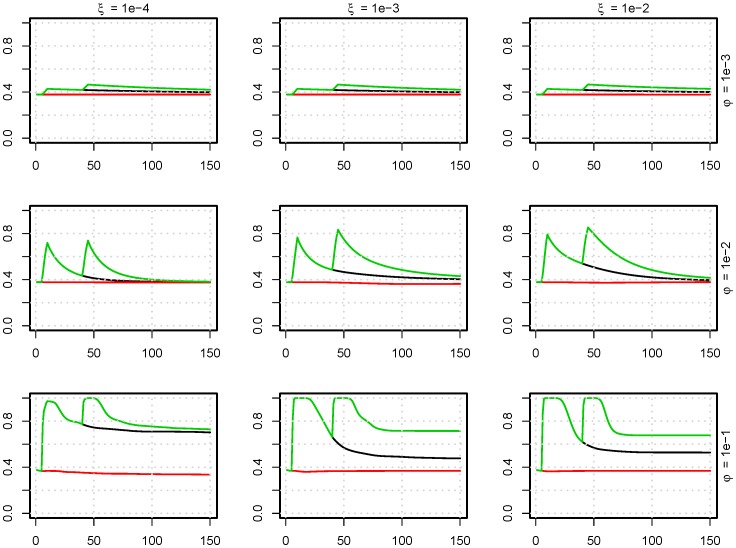
The influence of AMT on the level of resistant strains. Fraction of antimicrobial resistant bacteria (y-axes) as a function of time [days] (x-axes) with antimicrobial treatment. The time of AMT is indicated by color: red: no treatment, black: treatment from day 5 to 10, green: additional treatment day 35 to 40. This is modelled for nine parameter sets of 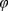 (excretion rate) and 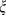 (uptake fraction from the environment). (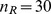).

**Figure 7:**
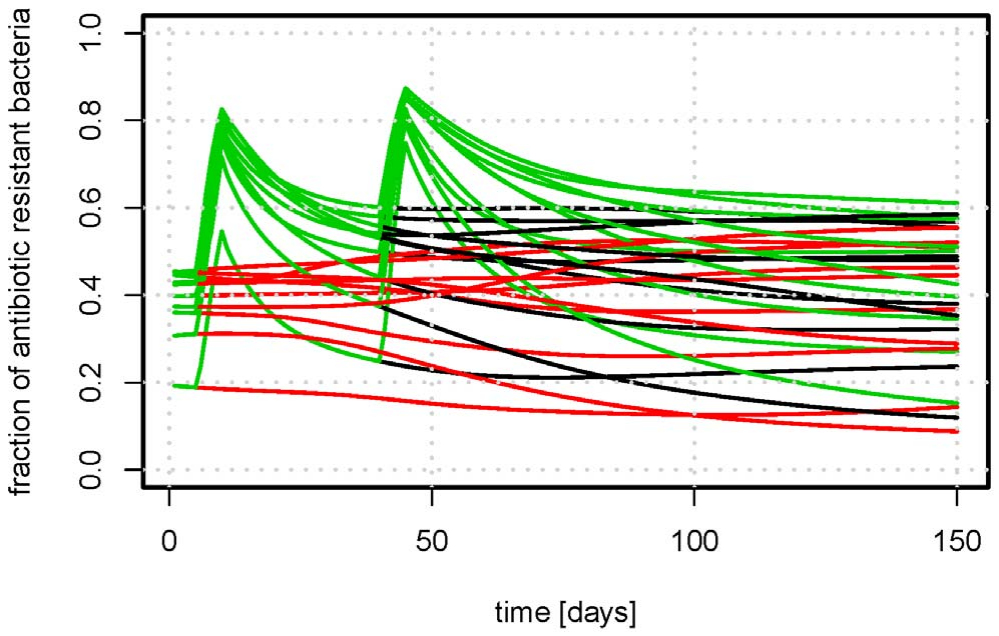
The variation of the repeats from figure 6. Fraction of antimicrobial resistant bacteria as a function of time with antimicrobial treatment. The AMT are indicated by color: red: no treatment, black: treatment from day 5 to 10, green: additional treatment day 35 to 40. The graph depicts 10 repeats with different compositions of initial strains. (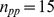, 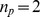, 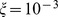, 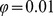).

Co-existence of multiple strains was achieved in our model across a wide range of parameter values, where we observe the system to enter an equilibrium state. When referring to equilibrium throughout this paper it is the co-existence of multiple strains in every pig, where only small changes happen over long periods of time, unless the system is disturbed by e.g. an antimicrobial treatment. After a disturbance the system will again return to a state of equilibrium, which may or may not differ from the original one. Such equilibrium situations are represented in figures 2 and 3, showing the dynamics of strains in four pigs with and without an antimicrobial treatment (AMT), respectively. The equilibrium in individual pigs resulted in equilibrium in the population, where we summarize over all bacteria in all pigs (figures 4, 5, 6, and 7). Equilibrium did not dependend on pigs having identical composition of strains. The equilibrium we observed was not stable over infinite time, and was as such not a strict mathematical equilibrium. However, because we observed stability over a timescale comparable to the lifetime of the pigs, we will use the term ‘equilibrium’ as this is the intuitive terminology for reaching a steady state.

Using the proposed model equilibrium in both pigs and the population was established for all realistic values of the uptake fraction from the environment, 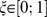, and number of strains, 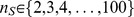. Equilibrium was possible, as long as the excretion rate was below the growth rates of the strains, so that the condition 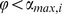 was met for more than one strain, 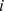, in the run. If 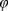 was larger than the growth rates, then the strains will all vanish with time (see File S1 for derivation of stable limits of the system).

The side-effect of an antimicrobial treatment was a growth advantage of resistant strains, which results in growth to high proportions in the individual pigs (figure 3). Moreover, the growth to high levels in the pigs resulted in increased transfer of resistant strains to pigs that did not have them previously.

Figure 4 displays how large a fraction of possible strains (i.e. 5, 10, or 20) that was present in the average pig; this is given by 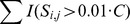 / 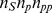, where 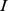 is the indicator function. For example, if the black line (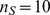, for 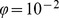, 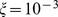) levels at 0.8, then there is on average 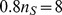 strains present in each pig at equilibrium. This also represent the level of homogenisation of the strains, the value 1 represent that all strains are present in all pigs.

Two principal domains in the figures 4–7 can be observed. One for low values of 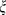 and 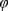 where there is no change in the composition of strains in the pigs, and consequently no deviation from initial values in the graphs. This first domain has such a small exchange of strains that the initial distribution of strains has very little change. The second domain for high values of 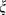 and 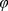 shows the strains transferring between pigs to an equilibrium state. When reaching equilibrium, there is not 100% homogenization (all possible strains are not present in all pigs), and the level of homogenization depends on all of the parameters. The effect of the uptake fraction from the environment, 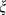, on the final level of equilibrium was small when 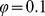, but it had larger effects when 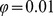. An increase in the uptake fraction from the environment, 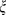, lead to the pigs reaching equilibrium faster and reaching a higher level of homogenization. An increase in the excretion rate, 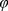, also decreased the time it took to get to the equilibrium state; if such a state exists. The level of the equilibrium was highest for medium values of 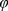 because the 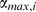
's were drawn from a distribution where some will be lower than the excretion rate, 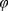, when it was high. Therefore, the fraction of strains in a pig did not reach 100% for high values of 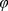 (See also figure 5). It was also apparent that increased competition in forms of higher diversity of bacterial strains, 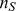, limited the possibility of presence/survival, which was defined as exceeding 1% of the population.

Note, that the decreased time to equilibrium in figure 4 when increasing 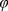 is due to both the intake, 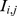 (eq. 5), increases with 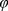, and that the equilibrium will be further from the carrying capacity, 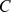, which allows for faster growth.

The influence of AMT on the model is presented in figure 6, where treatment is compared to no treatment. Here, the effects of 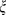 and 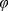 were also non-linear. Increasing 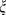 for 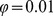, increased the period where resistant strains dominates. But increasing 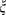 when 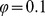 increased the speed at which the susceptible strains could re-establish themselves in the population. However, only increasing 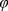 gave the resistant strains better growth opportunities during treatment, which lead to an increase of resistant strains in the population that could be observed for a long period of time after treatment. For all combinations of 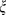 and 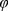, there were large variations in the outcome, as seen in figure 7. Repeated treatment increased the level of resistant bacteria at day 150 compared to one or no treatment.

**Figure 8:**
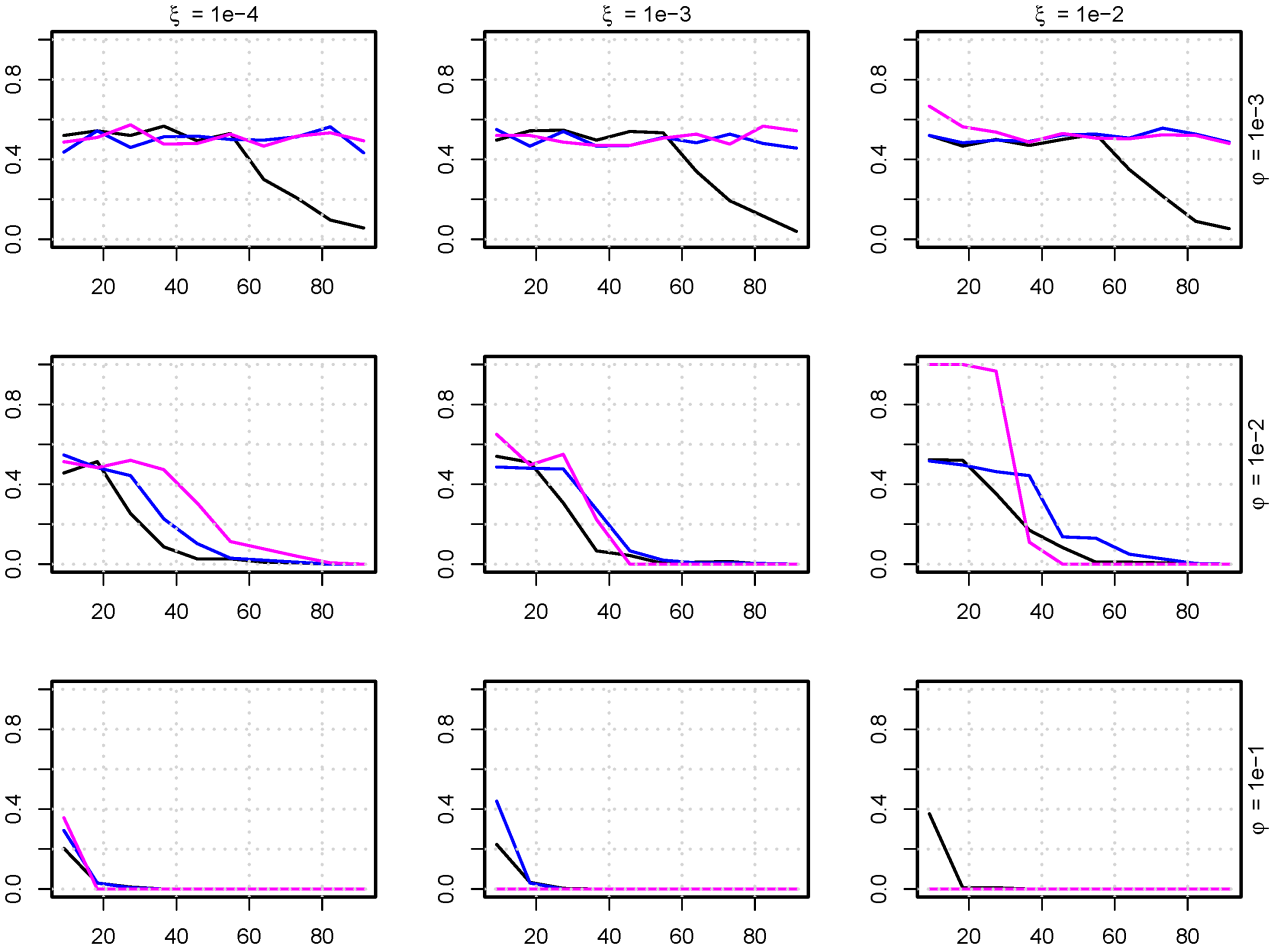
Survival of fitness reduced bacteria. The fraction of pigs that has survival of the strain (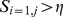) (y-axes) with a percentage reduction in growth rate compared to the mean growth rate (x-axes). The colors indicate the extinction cut off, 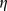, under which bacteria risk extermination from the pig: pink = 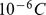, blue = 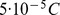, black = 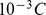, where 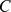 is the carrying capacity of the system (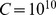). This is modeled for nine parameter sets of 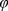 (excretion rate) and 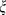 (uptake fraction from the environment). For example, for 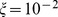, 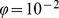 at all three cut off values, 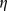, the (resistant) strains are only extinct when the fitness cost is approximately 40%. (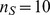, 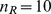).

In figure 8, the amount of reduction in fitness, described in terms of percentage reduced growth rate, a bacterial strain can have and still survive somewhere in the population depending on 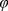, 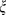, and 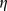 is shown. The larger the 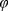, the harder it becomes to survive with a low growth rate. The uptake fraction from the environment, 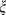, primarily influenced survival at the set (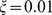, 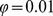), where high uptake fraction ensures a higher survival if the extinction cutoff, 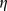, is low. The extinction cutoff, 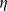, was most influential when the excretion rate was low, 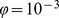; where a high 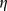 made it difficult for strains to survive. For all simulations where 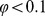 there was no change in the probability to survive as long as the fitness cost was below 20%.

Generally, increasing 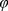 and 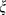 increased the variation in the outcome of the runs (not shown), which can be attributed to more transfer of bacterial strains between pigs, which increase the number of likely outcomes. The results presented are for the number of pigs per pen 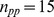, but 25 or 50 pigs in the pen were also tested, which gave near identical results (not shown). The reason for the similarity between the results of varying 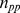 was likely due to the normalizing of equation 5. However, we would expect that if the number of strains surpasses the number of pigs per pen, 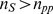, then the results would not be similar.

## Discussion

Our model simulates how multiple bacterial strains in multiple pigs may compete and spread. The model shows that the bacterial population will not crash or be overtaken by a single strain (‘prevail or perish’) but have coexistence of several strains, which includes strains with reduced growth rates, across all realistic parameter values. This is in concordance with our expectation of the composition of strains *in vivo* (c.f. Schierack et al. [21]). The total population of strains was also affected by antimicrobials that altered the transmission patterns of the strains, which has been reported in other studies [11], [12].

We have demonstrated that the transmission of bacteria between pigs influences the level of resistant bacteria in a population following antimicrobial treatment (figure 6). The transmission is described by four parameters, two that governs the deterministic transfer of bacteria between pigs, and two that determines the stochastic probability of surviving the transmission.

The two parameters that control deterministic transfer of bacteria are: the excretion rate of bacteria from the intestines of the individual pig to the environment, 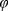; and the uptake fraction from the entire excreted material, 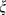. Both or these parameters may have both contributing and limiting impact on resistance spread: High excretion rate leads to faster bacterial spread in the pig pen, but it may also quickly eliminate strains with reduced growth rate. High uptake from the environment also leads to faster spread of bacteria within the pen, but facilitate a faster return of susceptible strains after end of antimicrobial treatment.

The two parameters that govern the probability of transfer of bacteria are: the probability of being removed within a given time interval, 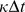, and the cut-off value under which this probability is enforced, 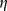. If these parameters are set low, then the simulation becomes deterministic; whereas if they are set very high no transmission of strains between pigs will occur. Given that adjusting these two parameters gives similar results (not shown), we have only shown the sensitivity of the model to one of the parameters (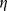 in figure 8).

The model presented has not been validated against data, except that the qualitative behavior of the system matches the reported behavior as cited above, e.g. Schierack et al. [21]. However, the model was built so that the parameters should be recognizable and identifiable to be tested by *in vitro* and *in vivo* experiments, e.g. growth rate experiments for individual strains or transmission trials with genetically marked strains.

In this study, the growth rates, 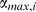, were reduced by a factor of 10 compared to unrestricted growth rates *in vitro* that have been reported in the literature [23]. This reduction was done to better reflect *in vivo* growth rates derived from animal experiments [19], [24], which describes models of a similar type to the one presented in this paper. However, this reduction in growth is perhaps already implicitly included, given that the carrying capacity limits growth when near the equilibrium, and therefore, the growth rates should possibly be as determined by unrestricted growth. The effect of an eventual increase in the magnitude of the growth rates is that the equilibrium is reached faster.

Our model predicted that the use of antimicrobials increase the level of resistant bacteria in a population, which is in concordance with the surveillance of Danish pigs [3] and other similar models [6], [15], [25]. We further demonstrated that besides the expected rise within-pig due to the growth of resistant strains during treatment, the increased levels of bacteria following treatment may also increase the transmission of resistant strains between pigs in a pen. Importantly, this was achieved without introducing special rates that are only in effect during treatment. The increase in transmission of resistant bacteria during treatment is due to the increased amount of resistant bacteria in the intestines; given that a constant fraction of all bacteria are excreted to the environment, an increase of resistance in the intestines leads to increased resistance bacteria in the environment, which lead to increase intake, and hence a greater probability of transmission between pigs.

The model presented in this paper differs from the very simple rate models by the double term including the carrying capacity, 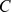, in equation (3). This double term is necessary as the strain with the highest growth rate will otherwise outgrow all other strains and equilibrium cannot be established. An interpretation of the terms may be that the total population, 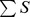, cannot exceed 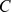 because the total amount of nutrition present is limited, and a single strain cannot utilize all the types of nutrients, and therefore must also be limited. Given the assumption that all strains are independent, we interpret this as strains having different nutritional niches or preferable location in the intestinal system.

Emergence or transmission of resistance between bacterial strains was not included in the model, because the level of resistant strains is high within the pig population [3], and so the growth and subsequent transmission of bacteria between pigs is a larger factor than conjugation or other means of transmission of resistance between strains. The rates of conjugation reported in the literature range between 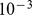 [day^−1^] [24] to 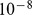 [26], depending on the reference volume of the carrying capacity. However, even in the high end of recombination rates, this is much smaller than the growth rate, which is of the order 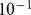. Given that resistant bacteria are present in high numbers, the growth of resistant bacteria will outnumber conjugation events more than one hundred to one.

The excretion rate, 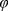, is considered to be constant throughout the course of time in the model. But we propose that 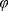 in reality could vary over time, e.g. diarrhea may be associated with higher values of 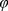. Diarrhea may originally have been a good strategy to diminish the number of strains in the pig gut, as a very high 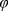 eradicates many of the non-dominant strains from the system, and hopefully allows the pig to maintain most of its original bacteria flora. However, when 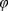 is high, a treatment with antimicrobials gives resistant strains additional advantages (figure 6) which aid the selection of resistant strains. Therefore, bacterial excretion via diarrhea seems a less beneficial strategy to the antimicrobial treated pig. Repeating treatments seems to be especially bad when the excretion rate is high, because this increases the probability of removing susceptible strains.

The uptake fraction from the environment, 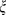, is varied between a re-uptake of bacterial strains of 0.01% and 1%; these limits were chosen to represent a wide range of situations. The amount of transmitted bacteria from faeces in the pen is most likely affected by many factors (i.e. the fraction of area of the pen with slatted floor, or the amount of hay in the pen). We reduce the factors influencing bacteria in the environment into one parameter: the fraction of the total excreted bacteria that is re-ingested by the pigs, 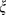. Hence, there is no explicit modelling of the behaviour of the bacteria outside the pigs, and the mode of transmission is formally direct transmission. This simplification leads to transmission being instantaneous; where *in vivo* it would be delayed by some hours. Given that *E. coli* has a relatively short lifespan outside the host [27] the simplification should not influence our conclusions.

We will now further discuss the assumptions made for this model:

Firstly, strains were fully identified and described by their growth rate and response to antibiotics. The motivation for this is that if we cannot differentiate bacteria, then they are the same strain. Since we are only modelling growth in response to antibiotics, we do not make conclusions based on e.g. clumping of bacteria, or adhesion to the intestinal walls.

Independence and uniqueness of the strains mean that we do not model interactions of the bacterial strains. Bacteria interact in many ways, and the most relevant interaction is that they exchange resistance, which we deal with in the next assumption. We have argued earlier that the exchange of resistance is rare compared to growth events. If bacteria have other traits that are not connected to growth or antimicrobial resistance, then the bacteria are not different for the purpose of this model, as expressed by the first assumption.

It was assumed that the pen level represents the maximum level of spread for bacterial strains. Modeling spread on section level may be more optimal when trying to evaluate experimental data. However, spread between pens may be dependent on actual pen layout, which was intentionally not included in the current model, so that the conclusions did not depend on specific section designs. Modeling of the spread between pens can be envisioned in multiple ways, such as a fixed rate between immediate neighbors or a distance dependent spread-kernel. Another spread mechanism may be the movement of pigs between pens. Pigs are usually kept in the same pen during the weaner period. However, some farmers will move weaker individuals to younger batches for a more consistent size. This practice may be a potential route of transmission. For simplicity this study has not considered the effect of different managerial practices.

Disease was not modeled explicitly, and any of the strains in the model can be thought of as a disease causing strain. As previously mentioned, an increased 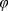 could be thought of as representing diarrhea, and the model could be executed with variable excretion rates as a function of the level of a selected strain. Linking a particular strain and excretion rate/disease was not done, as there is no good estimate of how this should be done, and the data already established that increased 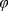 led to a higher transmission of strains.

In the model runs, no form of intervention other than antimicrobial treatment was regarded. This includes such events as introduction of other bacterial strains by people or new batches of pigs, and other treatments performed by veterinarians or farmers. These factors were not included, as they may be specific to a certain farm, veterinarian, or country. We did also not include any behavioural patterns of the pigs that may lead to other than complete random uptake of bacteria, because such patterns may depend on many factors that are local to the farm. The treatment protocol was assumed to result in a constant 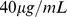 concentration of antibiotics in the intestines of the pigs. The true concentration will naturally vary with the drinking patterns of the pigs, which may introduce variation of the antimicrobial concentration. This assumption may therefore result in a slight overestimation of the growth advantage of the resistant strains, and reduce variation of the outcome.

Figures 5 and 7 reveal that the initial composition of strains and the stochastic transmission of strains leads to large variation in the equilibrium composition of the system. A large variation of bacterial resistance can also be observed *in vivo* (expert opinions and [3]), which emphasis the need for models which includes multiple strains, as the one presented here.

Whether repeated treatments raised the final level of resistant bacteria in the pen depended on both the uptake fraction and excretion rates (figure 6). When the uptake fraction and excretion rates were high, the probability that susceptible bacterial strains died out in competition with resistant strains increased with the period of time that the resistant bacteria were in advantage due to treatment(s) (figure 4). A comparison of figures 4 and 6 shows that when the final level of ABR bacteria was the same as the initial level (in particular for 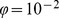 and 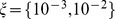), the number of resistant bacterial strains was higher.

This study noticeably showed that resistant strains with a high fitness (i.e. well over 10% reduction in growth rate) cost can survive in a small fraction of the pigs (figure 8). From these individual pigs, they may spread rapidly through the population if advantages, e.g. in the form of AMT, occur. Compared to models of resistant strains with a growth disadvantage, which are expressed as having a linear rate in the differential equations, the model presented in this paper was able to explain how resistant strains survive in the pig population through the equilibrium of multiple strains; even in the case of reduced fitness.

## Supporting Information

File S1 **Supplementary Material.** Examples of the equations for different number of strains and pigs, including derivations of stable limits of the system.
(PDF)
