# Supplementary material for: How Fitness Reduced, Antimicrobial Resistant Bacteria Survive and Spread: A Multiple Pig - Multiple Bacterial Strain Model
Source: PLoS One. 2014 Jul 9;9(7):e100458. doi: 10.1371/journal.pone.0100458 (PMC4090066; doi:10.1371/journal.pone.0100458)
Supplement: File S1 — Supplementary Material. Examples of the equations for different number of strains and pigs, including derivations of stable limits of the system. (PDF) [file pone.0100458.s001.pdf]

Supplementary Material - SM1.

How Fitness Reduced, Antimicrobial Resistant Bacteria Survive and Spread: A Multiple Pig - Multiple Bacterial Strain Model.

Kaare Græsbøll, Søren Saxmose Nielsen, Nils Toft, Lasse Engbo Christiansen

Mathematical appendix

We shortly restate the main equations given in the manuscript:

$$dS_{i,j}/dt = (G_{i,j} - E_{i,j} + I_{i,j}) \quad , \tag{1}$$

where $S_{i,j}$ denotes the bacterial count of the i'th strain of bacteria in the j'th pig's intestines; $G_{i,j}$ is the growth of bacteria; $E_{i,j}$ describes the excretion of bacteria to the pen environment; $I_{i,j}$ describes the intake of strains from other pigs; and t is time. Please note that S represents any strain of bacteria not only susceptible strains.

The competitive growth of strains in one pig:

$$G_{i,j} = H_{i,j}\ S_{i,j}\ \frac{(C - S_{i,j})\,(C - \sum_i S_{i,j})}{C^2} \quad , \tag{2}$$

where $G_{i,j}$ expresses the total growth term per strain per pig; and C is the bacterial carrying capacity of the intestines in each pig. The

$$H_{i,j} \equiv H(\alpha_{max,i}, \gamma_i, EC_{50,i}, c_j) = \alpha_{max,i}\left(1 - \frac{c_j^{\gamma_i}}{EC_{50,i}^{\gamma_i} + c_j^{\gamma_i}}\right) \quad , \tag{3}$$

where $\alpha_{max,i}$ is the growth rate of the i'th strain when no antimicrobial is present; c_j is the antimicrobial concentration in the j'th pig; $EC_{50,i}$ is the antimicrobial concentration at which the bacteria grow at half the maximum rate, $\alpha_{max,i}$; and γ_i is the 'hill-coefficient', which determines the steepness of the curve around $EC_{50,i}$.

The excretion of strains from the pigs' intestines is described by:

$$E_{i,j} = \varphi S_{i,j} \quad , \tag{4}$$

where φ is the rate at which bacteria is excreted from the intestines.

The intake of strains from other pigs in the pen is defined as:

$$I_{i,j} = \frac{\xi}{n_{pp}} \sum_j E_{i,j} = \frac{\xi\varphi}{n_{pp}} \sum_j S_{i,j} \quad , \tag{5}$$

where ξ is the fraction of bacteria that comes back in from the environment. The environment is defined by the combined excretion from the pigs that share a pen. The equation is normalized by the number of pigs per pen, n_{pp}, so that the intake of feces does not increase with an increased pen size.

Removal, $R_{i,j}$, of a bacterial strain, i, from the j'th pig is an event described by the probability:

$$P\left(R_{i,j} \in [t; t + \Delta t] \mid S_{i,j} < \eta\right) = \kappa\Delta t \quad , \tag{6}$$

so that there is a probability $\kappa\Delta t$ that the strain $S_{i,j}$ becomes zero within a given time interval, $[t; t+\Delta t]$, given that the bacterial count, $S_{i,j}$, is below η. This term can be thought of as the probability of surviving in the gut when entering from the external environment, or losing the competition to strains with higher growth rates.

In the following we will derive analytical results for reduced models to exemplify and clarify the results of the full model. Notice that for this appendix the equilibrium as defined in the paper becomes equivalent to the stationary state of coupled ordinary differential equations as the probability of removal is left out.

One pig, two strains

For simplicity we assume that we have only one pig, two strains, and no antimicrobial treatment. In this setting equation (1) becomes:

$$\begin{aligned} dS_1/dt &= \alpha_1\, S_1\, \frac{(C-S_1)\,(C-(S_1+S_2))}{C^2} - \varphi(1-\xi)S_1 \\ dS_2/dt &= \alpha_2\, S_2\, \frac{(C-S_2)\,(C-(S_1+S_2))}{C^2} - \varphi(1-\xi)S_2 \end{aligned}$$

where we have omitted the *max* subscript on α. We see immediately that if the sum of strains S_1+S_2 is equal to the carrying capacity, C, then no growth will occur, which justifies the term carrying capacity.

We are interested in finding stationary solutions and thus want to solve $dS_i/dt = 0$ we define $S_i = \lambda_i C$, $\sum_i S_i = \Lambda C$, and $\phi = \varphi(1-\xi)$. If we limit the initial states to fulfill $\Lambda \in [0;1]$, the two trivial solutions are for $\Lambda = 0$ and $\Lambda = 1$ ($S_i = 0\ \forall\ i$ and $\sum^i S_i = C$). However, these two solutions are not stable, and we must look for a solution were growth and excretion of the strains balance:

$$\begin{aligned} \alpha_1(1-\lambda_1)(1-\Lambda) = \quad \phi \quad &= \alpha_2(1-\lambda_2)(1-\Lambda) \quad \Rightarrow \\ \alpha_1(1-\lambda_1) \quad &= \quad \alpha_2(1-\lambda_2) \end{aligned} \tag{7}$$

introducing the relative growth rate, θ, as $\alpha_1 = \theta\alpha_2$ leads to:

$$\begin{aligned} \lambda_2 &= 1-\theta+\theta\lambda_1 \\ \Lambda &= 1-\theta+(1+\theta)\lambda_1 \end{aligned}$$

and inserting into equation (7):

$$(\theta+1)\lambda_1^2 - (2\theta+1)\lambda_1 + \left(\theta - \frac{\phi}{\theta\alpha_2}\right) = 0$$

This equation has the discriminant:

$$d = 4\frac{\phi}{\alpha_2}\left(\frac{1}{\theta}+1\right)+1$$

which is always positive for $\theta \in [0;\infty[$. The extremum of the parabola is located at:

$$T = \frac{2\theta+1}{2(\theta+1)} = 1 - \frac{1}{2(\theta+1)}$$

From this it can be observed that the extremum can only be $T \in [0.5;1]$. To guarantee that there is only one root of λ_1 in the interval $]0;1[$ is equivalent to:

$$\begin{aligned} \frac{\sqrt{d}}{2(\theta+1)} &> 1-T = \frac{1}{2(\theta+1)} \quad \Leftrightarrow \\ \sqrt{d} &> 1 \end{aligned}$$

which is true for all $\theta \in [0;\infty[$. Therefore, the equation have zero or only one root in the interval $]0;1[$. The lower limit for having one root in the interval $]0;1[$ is $\theta = \sqrt{\phi/\alpha_2}$. For typical values of $\phi = 0.01$, $\alpha_2 = 0.1$, and $\theta = 2$, this gives $\widehat{\lambda}_1 = 0.62$ and $\widehat{\lambda}_2 = 0.25$, notice that $\widehat{\lambda}_1 + \widehat{\lambda}_2 < 1$, and $dS_i/dt < 0$ when $\lambda_i > \widehat{\lambda}_i$ and all other λ_k are kept at the stationary solution ($\lambda_k = \widehat{\lambda}_k$). If the equilibrium is such that $\widehat{\lambda}_i C < \eta$ then the strain will eventually be removed by the removal probability.

One pig, multiple strains

It is not trivial to derive the equilibrium conditions with more than two strains. However as observed previously the system has unstable equilibria at $\Lambda = 0$ and $\Lambda = 1$, and given that the system is of second order, any equilibrium found within this region must be stable. The differential equations can be described as limiting growth progressively the closer the system is to the carrying capacity. Whether the system will have an equilibrium or not depends on the growth rates of the strains. Some values of growth rates will lead to equilibrium conditions below the cutoff, the strains with these growth rates will die out. The condition on when strains will die out can be assessed by defining

$$\beta_i = \frac{(C - S_i)\left(C - \sum_i S_i\right)}{C^2}$$

If the system is initialized with $\sum_i S_i < C$, it is evident that β_i can only be in the interval $[0;1[$, because $\beta \to 0$ for $\sum_i S_i \to C$. Now $dS_i/dt < 0 \Leftrightarrow \alpha_i \beta_i < \varphi(1-\xi)$ which is the definition of negative growth, since β_i has an upper limit of one, the growth rate, α_i, must be larger than $\varphi(1-\xi)$ for the strain to be able to increase in numbers.

Two pigs, two strains

We write the full set of equations for two pigs with two strains:

$$\begin{aligned}
dS_{1,1}/dt &= \alpha_1\, S_{1,1}\, \frac{(C - S_{1,1})\,(C - (S_{1,1} + S_{2,1}))}{C^2} - \varphi\left(1 - \frac{\xi}{2}\right) S_{1,1} + \frac{\varphi\xi}{2} S_{1,2} \\
dS_{2,1}/dt &= \alpha_2\, S_{2,1}\, \frac{(C - S_{2,1})\,(C - (S_{1,1} + S_{2,1}))}{C^2} - \varphi\left(1 - \frac{\xi}{2}\right) S_{2,1} + \frac{\varphi\xi}{2} S_{2,2} \\
dS_{1,2}/dt &= \alpha_1\, S_{1,2}\, \frac{(C - S_{1,2})\,(C - (S_{1,2} + S_{2,2}))}{C^2} - \varphi\left(1 - \frac{\xi}{2}\right) S_{1,2} + \frac{\varphi\xi}{2} S_{1,1} \\
dS_{2,2}/dt &= \alpha_2\, S_{2,2}\, \frac{(C - S_{2,2})\,(C - (S_{1,2} + S_{2,2}))}{C^2} - \varphi\left(1 - \frac{\xi}{2}\right) S_{2,2} + \frac{\varphi\xi}{2} S_{2,1}
\end{aligned}$$

The difference between a single and multiple pigs are the intake of bacterial strains from the environment, which is defined as excretion from all the pigs.

Again it is not trivial to determine the exact equilibrium of the system. It is, however, still apparent from the equations that a bacterial count above the carrying capacity, C, gives a negative contribution to the growth rate, which contain the population. While the lower unstable limit is now that both pigs do not possess the i'th strain ($\sum^j S_{i,j} = 0$). There is therefore still the possibility of a stable equilibrium existing within $\lambda_i \in]0;1[$ ($S_i \in]0;C[$).

Multiple pigs, multiple strains

With the same arguments as above, there is a possibility of stable equilibrium for the i'th strain in the j'th pig if $\alpha_i \beta_{i,j} = \varphi(1 + \xi/n_{pp} \sum_k S_{i,k}/S_{i,j})$ can be fulfilled. When there are multiple pigs the strains get help from the strains in other pigs in the pen to survive. Notice if all pigs have the same number of bacteria i, $S_{i,k} = S_{i,j}$ for all k, then the condition is equivalent to when there is just one pig.

All this has been in the absence of treatment(s). The overall effect of treatment is a shift of the population towards strains that can grow while treated. After a treatment the population will converge towards the untreated equilibrium if left untreated for sufficiently long time, and if no susceptible strain is totally removed from the population.

Removal probability

So far we have not discussed the removal of strains that are present only in low numbers, as described by equation (6). The parameters governing this are: the probability of being removed within a given time interval, $\kappa\Delta t$, and the cutoff value under which this probability is enforced, η. If these parameters are set low the simulation becomes deterministic, because this would subject less bacteria to stochastic events. However if they are set very high no transmission of strains between pigs will occur.

In the derivations previously presented in this appendix no stochastic elements were imposed. However, the derivations still hold if the parameters is set low enough that enough bacteria is transferred to surpass the threshold, η. In case all strains are initially present in all pigs only strains that will have equilibrium below η are at risk of removal.

The effect of imposing a threshold has the consequence that when there are many pigs in the population, $n_{pp} \gg 1$, one strain present in only one pig, will not quickly colonize all pigs within the pen, as the fraction of bacteria in the environment of this strain is small, and so this strains will not easily surpass the removal probability. If this stochastic approach is not implemented then all strains will immediately be present in all pigs after the start of the simulation and all pigs will converge towards the same stationary solution. Moreover, strains with high growth rates will extremely fast become dominant of the total population.
